# Evaluation of the Effectiveness of a Whole-System Intervention to Increase the Physical Activity of Children Aged 5 to 11 Years (Join Us: Move Play, JU:MP): Protocol for a Quasiexperimental Trial

**DOI:** 10.2196/43619

**Published:** 2023-03-31

**Authors:** Daniel D Bingham, Andy Daly-Smith, Amanda Seims, Jennifer Hall, Lucy Eddy, Zoe Helme, Sally E Barber

**Affiliations:** 1 Faculty of Health Studies University of Bradford Bradford United Kingdom; 2 Centre for Applied Education Research Wolfson Centre for Applied Health Research Bradford Royal Infirmary Bradford United Kingdom; 3 Bradford Institute for Health Research Bradford Teaching Hospitals NHS Foundation Trust Bradford Royal Infirmary Bradford United Kingdom

**Keywords:** physical activity, accelerometry, complex intervention, whole system, children, quasiexperimental

## Abstract

**Background:**

Daily physical activity is vital for the health and development of children. However, many children are inactive. Previous attempts to achieve sustained increases in daily physical activity in children have been ineffective. Join Us: Move Play (JU:MP) is a whole-system, complex, community-based intervention aiming to increase the physical activity levels of children aged 7 to 11 years who live in areas of Bradford, England, which are multicultural and have high levels of deprivation.

**Objective:**

The purpose of this quasiexperimental controlled trial is to assess whether the JU:MP program increases primary school children’s physical activity.

**Methods:**

The study has a 2-arm, quasiexperimental, nonblinded, nonequivalent group design and will be conducted with primary school children aged 5 to 11 years at 3 timepoints, including baseline (before intervention), 24 months (during intervention), and 36 months (after intervention). Children attending primary schools within the intervention area will be invited to participate. Children attending similar schools within similar neighborhoods based on school and community census demographics (deprivation, free school meals, and ethnicity) outside of the JU:MP geographical area will be invited to participate in the control condition. At each timepoint, consenting participants will wear an accelerometer for 7 consecutive days (24 hours a day) to measure the primary outcome (average daily moderate-to-vigorous physical activity). Multivariable mixed effects linear regression will be applied to estimate differences in the primary outcome between the 2 arms at 24 months and 36 months on an intention-to-treat basis. The secondary outcome analysis will explore changes in socioemotional well-being (teacher reported), quality of life (parental/carer reported), and other contextual factors (parents/carer reported), as well as segments of the day activity, sleep, sedentary screen time, frequency of places to be active, parent practices (nondirective support and autonomy support), social cohesion, and neighborhood walking/exercise environment.

**Results:**

Recruitment occurred from July 2021 to March 2022, and baseline data were collected from September 2021 to March 2022. As of March 2022 (end of baseline data collection), a total of 1454 children from 37 schools (17 intervention schools and 20 control schools) have been recruited. The first follow-up data collection will occur from September 2023 to March 2024, and the second and final follow-up data collection will occur from September 2024 to March 2025. Data analysis has not begun, and the final results will be published in December 2025.

**Conclusions:**

This article describes the protocol for a quasiexperimental controlled trial examining a novel whole-system intervention.

**Trial Registration:**

ISRCTN ISRCTN14332797; https://www.isrctn.com/ISRCTN14332797

**International Registered Report Identifier (IRRID):**

DERR1-10.2196/43619

## Introduction

### Background

Daily physical activity, particularly of moderate-to-vigorous intensity, is very beneficial for the physical, emotional, and social development of young people [1,2], and is preventive for the early onset of diseases and disorders [3-5]. This importance is enhanced when considering physical activity behaviors tracked through childhood into adulthood [6-9]. All 4 nations of the United Kingdom, along with the World Health Organization, recommend that children aged 5 to 18 years should engage in moderate-to-vigorous–intensity physical activity for an average of 60 minutes a day [10]. Since the publication of government guidance, evidence has shown that engaging in daily light-intensity physical activity [11] or increasing daily moderate-to-vigorous activity by an average of 10 minutes a day [12] can be beneficial for children and young people.

Worryingly, levels of inactivity are high across the globe [13,14]. Much effort has been made to combat levels of inactivity among young people [15,16], but previous interventions have been generally unsuccessful, especially in the medium to longer term [16,17]. The reasons for the lack of success so far include poor study design, poor methodology, and not considering the full complexity of physical activity, which is a behavior with much variance and heterogeneity across different groups and populations [16,17]. Previous interventions have only generally been discrete, simple, and 1-dimensional [16,17], although it has been over 3 decades since McLeroy et al [18] proposed that health promotion interventions and policies should consider every aspect of society from the social to physical environment, to individual personal traits. This socioecological perspective theorizes that change in such a complex behavior is not likely to occur just through individual motivation and desire alone, but instead through influencing every aspect of the socioecological model and wider system of society, to seek and promote more human movement. The challenge to decrease physical inactivity among all members of society, particularly children and young people, is only going to be addressed if future interventions and programs have a *systems thinking philosophy* and fully embrace the whole socioecological model within intervention and program planning and implementation [19]. The International Society of Physical Activity and Health supports this perspective with the publication of “Eight investments that work for physical activity,” which advocates whole-system change across 8 domains, including schools, communities, travel, urban design, health care, workplaces, mass media, and sports and recreation [20].

In response to the growing realization of a need for whole-system approaches to physical activity, Sport England (a nondepartmental public body, which is the largest funder of physical activity and sport programs in England) funded 12 local delivery pilots over a 5-year period (2019-2024), to design and implement a whole-system place-based approach to reduce physical inactivity and health inequalities. All of the selected pilot areas are characterized by high levels of deprivation, but target different populations (eg, older people and whole communities), with the Join Us: Move Play (JU:MP) program being delivered in Northern areas of the City of Bradford, targeting children and young people aged 5 to 14 years. Bradford is the 7th largest metropolitan district in England and is home to more than half a million people. A quarter of Bradford’s population is under the age of 16 years, making Bradford the “youngest” city in the United Kingdom [21,22]. Bradford is a culturally rich and ethnically diverse city with over 40% of children and young people being of South Asian heritage [23]. However, Bradford (city area) falls in the most deprived quintile of the index of multiple deprivation (IMD), with 60% of the population living in the poorest 20% of wards in England and with 24% of children living in poverty [21,22]. Given the high childhood population in Bradford and given that there is an inverse association between physical activity levels and age during childhood [24], successfully increasing physical activity for the child population in Bradford could have far reaching public health positives for the city in the future. For this reason, the JU:MP program is focused on increasing physical activity in the 27,000 children and young people aged 5 to 14 years and their families living in the area. This article details a protocol for the effectiveness evaluation of the JU:MP program and is part of a wider evaluation plan (including process evaluation – development, implementation, and evaluation), details of which have been described previously [25].

### Aims and Objectives

The aim of this quasiexperimental trial is to explore whether JU:MP is effective at increasing physical activity among children aged 5 to 11 years. The specific research objectives are as follows:

1. Determine the effectiveness of JU:MP to increase device-measured (accelerometry) mean average daily moderate-to-vigorous physical activity among children aged 5 to 11 years 24 months and 36 months after the implementation of a whole-system community intervention.
2. Determine the effectiveness of JU:MP to improve secondary outcomes among children aged 5 to 11 years 24 months and 36 months after the implementation of a whole-system community intervention. The secondary outcomes are as follows: (1) mean average daily, weekday, and weekend day sedentary time (device measured [accelerometry]); (2) mean average daily, weekday, and weekend day light physical activity (device measured [accelerometry]); (3) weight status and body fat level (waist circumference and BMI); (4) socioemotional well-being, including prosocial behavior, emotional problems, behavioral problems, peer problems, and hyperactivity/inattention (teacher reported); and (5) children’s quality of life (parental reported).
3. Undertake exploratory subgroup analysis (ie, neighborhood level and school level) investigating the change in the primary and secondary outcomes.
4. Investigate the potential meditating effects of changes in contextual factors (JU:MP is seeking to change; parental reported): (1) home physical activity, (2) school time physical activity, (3) children’s sedentary screen time behaviors, (4) active travel and street physical activity, (5) parent practices (nondirective support and autonomy support), (6) neighborhood social cohesion, (7) neighborhood walking/exercise environment, (8) religious setting physical activity, (9) physical activity during sports and recreation, and (10) park/green space physical activity.

## Methods

### Intervention: JU:MP Program

A more comprehensive description of the JU:MP program can be found in the report by Hall et al [25]; a brief summary is contained herein.

**Figure 1:**
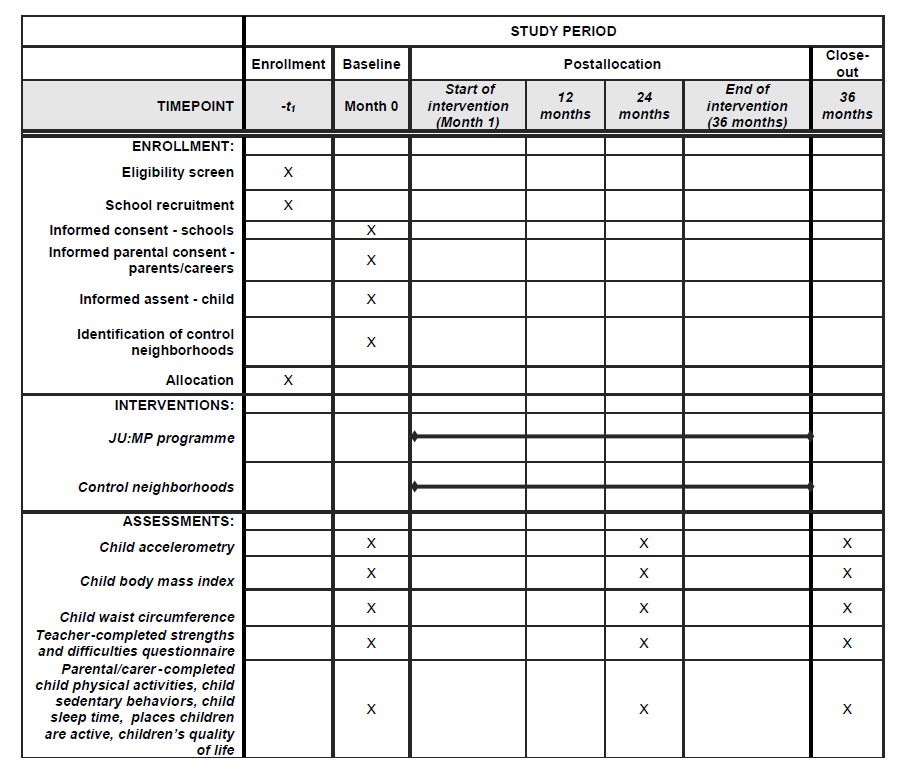
SPIRIT (Standard Protocol Items: Recommendations for Interventional Trials) diagram illustrating the design and timescales of the JU:MP controlled trial. JU:MP: Join Us: Move Play.

**Figure 2:**
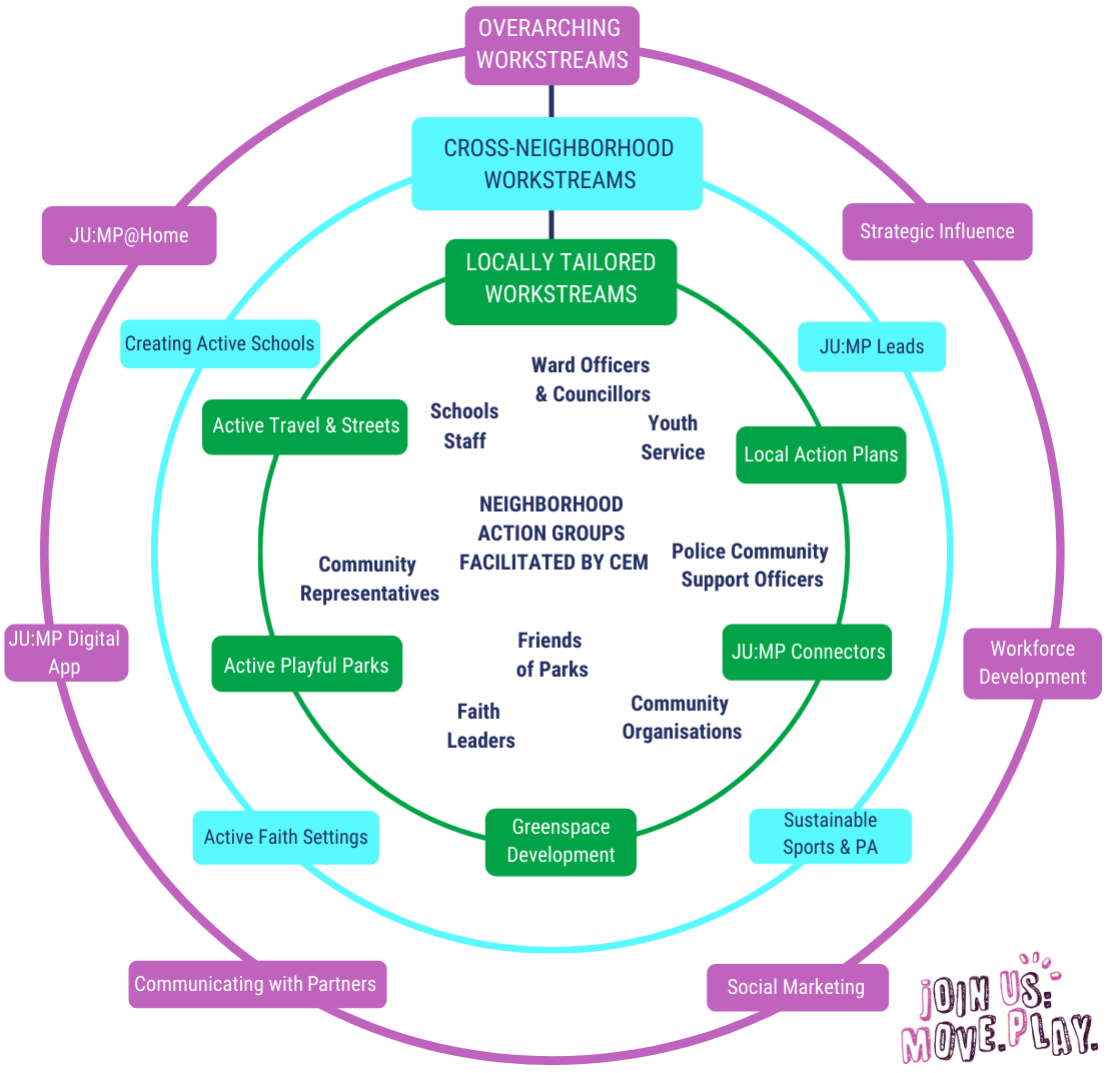
Join Us: Move Play (JU:MP) program model. CEM: community engagement manager; PA: physical activity.

The JU:MP program (Figure 1 outlines the SPIRIT [Standard Protocol Items: Recommendations for Interventional Trials] schedule [trial design and timescales]) is a whole-system, complex, community-based intervention that was originally developed in 2018 with underlying themes, a framework (settings, tools, and principles), and a theory of change being informed by community consultations, community priority setting workshops, data from the Born in Bradford research program [26-28], international peer-reviewed evidence [29,30], and the socioecological model [18,31]. After a period of development and piloting (2019-2020), the JU:MP program model was developed (Figure 2) [25]. The JU:MP program includes 15 interacting work streams, which include a focus on, for example, embedding physical activity in policy, parks and green spaces, communications and social marketing, and organizations including schools and faith settings. The program is being implemented within 8 distinct geographic “neighborhoods” within the Bradford local delivery pilot area (see Multimedia Appendix 1 for a map of the local delivery pilot neighborhoods). Neighborhood boundaries were based on having an area of green space with potential for development, at least 4 to 5 primary schools, and an active community organization. This hyper-local scale of whole-system delivery aims to foster genuine collaborative working and build strong sustainable relationships. Using an asset-based community development approach, JU:MP facilitates the development of an action group within each neighborhood, including key organizational partners, community members, and families. To allow the program to meet local needs and facilitate longer-term behavior change, the action group is responsible for designing and implementing change at the local level. Initially, the neighborhood approach was operationalized within 3 “pioneer neighborhoods” (2019-2021) to pilot and develop the program. Subsequently, the program will be delivered in the 5 remaining neighborhoods (2021-2024) to cover the whole local delivery pilot area. The remaining 5 neighborhoods are further broken down into those that are directly facilitated by the JU:MP team (n=3) and those whose delivery will be externally commissioned (n=2). Differences between implementation models are to permit examination of how JU:MP could be implemented differently in the future in different communities. The process evaluation outlined by Hall et al [25] will explore such differences; for the purposes of the trial described in this protocol, only effectiveness will be examined in the directly facilitated neighborhoods. The reason for this is a mixture of research capacity and a focus on the effectiveness of the fully developed JU:MP program.

### Design

**Figure 3:**
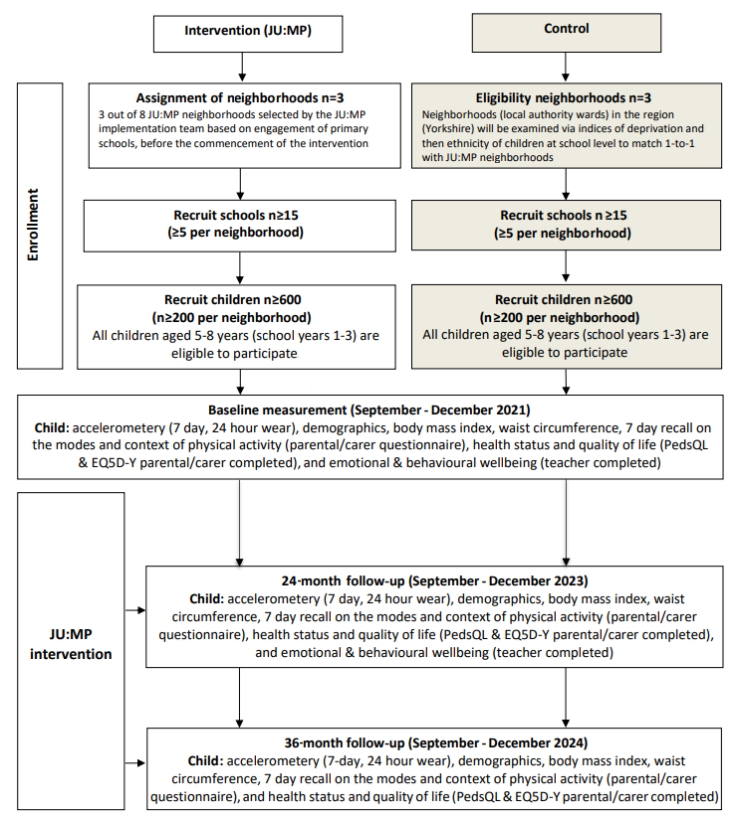
Join Us: Move Play (JU:MP) program neighborhood, quasiexperimental, nonequivalent group trial. EQ-5D-Y: EuroQol 5-dimension youth version; PedsQL: Pediatric Quality of Life Inventory.

The study has a 2-arm (intervention [JU:MP] and control), quasiexperimental, nonblinded, nonequivalent group design with 3 waves of data collection (baseline [before intervention], 24 months [during intervention], and 36 months [after intervention]). Figure 3 outlines the study flow diagram.

### Setting

The research will be situated within primary schools located across the Northern English County of West Yorkshire. Recruitment and data collection are taking place through primary schools as they are located within JU:MP neighborhoods, and children tend to live in close proximity to the school they attend. Primary schools allow access to a large number of children as potential participants, and previous research has shown that it is feasible and acceptable to conduct physical activity trials through primary schools [32].

### Identification of Neighborhoods

The JU:MP program is operating in 8 distinct neighborhoods. The 3 neighborhoods eligible for participation in this effectiveness trial are the 3 direct-delivery neighborhoods of JU:MP. Control neighborhoods will be based on local authority wards (local areas with elected officials representing the local area) and be in the county of West Yorkshire in order to ensure that neighborhoods would be politically and geographically similar to the JU:MP neighborhoods. Other neighborhoods/wards in Bradford have been excluded due to potential intervention contamination. Control neighborhoods must ideally have similar characteristics to the JU:MP neighborhoods to maximize internal validity. The process of identifying control neighborhoods will include pooling state-funded primary school census data [33], which are released from schools annually, for every ward in West Yorkshire and then calculating the median/mean of (1) the value of the school postcode-derived IMD (IMD 2019) [34]; (2) the proportion of children eligible for state-funded free school meals (school population measure of deprivation); and (3) the proportions of the 2 predominant ethnic groups in the 3 JU:MP areas (White British and South Asian heritage [combining Pakistani heritage, Bangladesh, Indian, and other South Asian ethnic groups]). Ward areas deemed to be suitable control areas must have no more than 1 IMD decile median difference than a JU:MP neighborhood and no more than 10% difference in the proportion of children eligible for free school meals, and the predominant ethnic group (majority or large minority) must be the same as that in a JU:MP neighborhood with ideally no more than 15% difference.

### Recruitment of Schools

Once the neighborhoods are selected, government-funded primary schools located in the JU:MP and control neighborhoods will be invited to participate in the research. The number of control schools recruited will be dependent on the recruitment of intervention schools in the neighborhoods to the study. Private and designated special schools have been excluded from this study. Identified schools will be contacted via standardized emails and follow-up phone calls (48 hours later) to request and arrange an in-person/virtual meeting to discuss the study and school-level consent. The primary schools in the JU:MP neighborhoods will be part of the JU:MP program and will receive whatever benefits may be derived from participating in the program. The primary schools within the control areas will not be offered access to the JU:MP program and must agree in principle not to commit to any whole-system school physical activity research in the next 3 years. To incentivize such a commitment, each of the primary schools in the control areas will be offered a total of £600 (US $727), with the caveat that this should not be spent on physical activity provision (physical education, active travel, after school clubs, etc) for the duration of the 3-year study. The £600 (US $727) payment will be split into 3 instalments of £200 (US $242) to be paid after the competition of each of the 3 study data collections (baseline, 24-month follow-up, and 36-month follow-up). If the school agrees to participate in the study, arrangements will be made to begin recruiting children.

### Recruitment of Children

All children in years 1, 2, and 3 (ages 5-8 years) at recruited schools will be invited to participate. Members of the research team will agree with school leadership on how best to speak and discuss about the study with all children in the school, but this will likely involve a researcher speaking to each class individually or the year groups collectively during a school assembly. Children will be provided with printed study information sheets and consent forms to take home for their parents/carers. The research team will ensure all forms are distributed to each class teacher and forms are sent home with children in book bags or school planners/diaries so that parents/carers can make an informed decision about whether their children can participate in the study. To ensure parents/carers have an opportunity to discuss the study with the research team, a time and day when they can attend a presentation and a question-and-answer session (within the school or virtually) will be arranged within a week of the information sheets and consent forms being issued. The research team will also seek to agree on times and days when researchers can be present in the school playground before and after school (pick up and drop off time). Short videos will also be produced, which will explain the study, and schools will be asked to distribute links of the videos (ie, YouTube) to parents. For children to participate, their parents/carers must provide informed consent. On the day of data collection, 2 members of the research team will discuss the study with each child and obtain verbal assent. For children with special educational needs/disabilities and those who do not have the capacity to provide assent, the research team will instead seek assent from a school staff member familiar to the child, with the child present. All children participating will receive a gift “goodie bag” worth an estimated £2 (US $2.42), which will include nonphysical activity–related items such as a book, stickers, and other similar items. The goodie bag will be given to each child at each data collection timepoint (baseline, 24 months, and 36 months).

### Materials

#### Primary Outcome Assessment: Accelerometry

The primary outcome assessment of this study will involve waist-worn accelerometry. At each timepoint, researchers will support children to fit an accelerometer (ActiGraph) worn on a belt around the child’s waist, just above the right hip. Children will receive a demonstration by a member of the research team, showing them how to fit the accelerometer. Children will be asked to wear the accelerometer for 7 consecutive days (24 hours a day; only to be removed for showering, bathing, and swimming). Children will be told that if it is uncomfortable to sleep in, they can remove it for sleep and wear it when they wake-up. ActiGraph data will be analyzed using ActiLife v6 (ActiGraph) and downloaded in 60-second epoch files. Sleep will be removed using validated sleep period–detection algorithms [35,36] (requiring 60-second epochs for this process) to produce sleep start and sleep end times. To ensure consistency with the chosen calibration of cut points and therefore increase validity [37], data will be reprocessed using 15-second epoch data in order to correspond with Evenson cut points [38] to classify time spent being sedentary, in light physical activity, and in moderate-to-vigorous physical activity. Sleep time will be removed by treating the sleep start and end times as sleep diaries within ActiLife. Nonwear time will be defined as 20 minutes of consecutive zeros and will be removed from the data. A valid day for inclusion in the analysis will be a minimum of 600 minutes, and children with at least 3 days of wear time, including at least 1 weekend day of data, will be included in the analysis. This wear-time criterion has been calculated using local Bradford data and has an estimated intraclass correlation of 0.75 [26].

#### Child Demographics and Anthropometric Data

After the receipt of parental/carer consent, researchers will request school-stored demographic data, such as date of birth, unique pupil number (linked to health records), biological sex, ethnicity, home postcode (for IMD calculation), child’s disability or special educational needs, and receipt of government-funded free school meals, from participating schools. The immediate request is to ensure the research team can plan provisions for any children with any additional needs during testing. On the first day of data collection, the research team will ask schools to provide the most recent attendance records and educational attainment data, and a copy of the class timetable for 2 weeks starting from the date of data collection (for accelerometry data collection). Anthropometric measurements will include weight, height, BMI, and waist circumference; all will be measured by trained researchers within the school setting. Weight will be assessed barefoot and in light clothing using a digital scale (eg, Tanita body composition analyzer SC-240MA III). Height will be measured unshod, with the head placed in the correct position, on a Seca 213 stadiometer (graduation=1 mm). BMI will be calculated and converted to a BMI percentile and z-score based on UK reference data [39]. Waist circumference will be measured using a Seca 201 tape (graduation=1 mm).

#### Children’s Socioemotional Well-being (Teacher Rated)

In order to measure children’s socioemotional well-being, class teachers of the recruited children will be asked to complete an assessment of the child’s behavior and socioemotional development using the strengths and difficulties questionnaire (SDQ) [40]. The SDQ is a short questionnaire (25 items) on positive attributes of the child as well as difficulties. The items are grouped into the following 5 subscales: “prosocial behavior” (ie, being helpful), “emotional problems” (ie, unhappiness), “behavioral problems” (ie, conduct problems), “peer problems” (ie, friendless), and “hyperactivity/inattention” (ie, restless). The SDQ teacher version has been found to have acceptable validity for evaluating psychosocial functioning in children [41].

#### Physical Activity, Sedentary Behavior, Sleep, and Quality of Life (Parent/Carer Rated)

On the day of data collection, children will be given a questionnaire to give to their parents/carers, which takes an estimated 20 minutes to complete. Parents/carers will complete this questionnaire at all 3 timepoints. Parents/carers will be asked to complete the questionnaire about their children’s life, rather than asking the children themselves, due to the young age of the children at baseline (5-8 years), which limits questionnaire comprehension and increases the risk of social desirability bias. The purpose of collecting such questionnaire data is to attempt to understand potential mediating factors of children’s accelerometer-measured physical activity. The content aligns with key components of JU:MP. This questionnaire has been piloted with groups of parents/carers living in West Yorkshire and has been deemed acceptable. The questionnaire comprises 6 sections. The first section relates to personal information of the children, including their name, school class, teacher’s name, age, and relationship with the person completing the questionnaire. The second section is the youth activity profile (YAP) [42], which is a published child-answered questionnaire. However, for this trial, parents/carers will be required to complete the questionnaire (due to the young age of the children), which entails reporting the frequency and duration of physical activities engaged in through segments of a usual day (ie, before school, break time at school, lunch at school, and after school). The YAP is also used to estimate sedentary behaviors of the children while watching television, playing video games, using a mobile phone, and using a computer/tablet during the previous 7 days. The third section asks parents/carers to report the normal time their children go to bed and wake-up on weekdays and weekend days, in order to calculate average sleep time [43]. The fourth section asks questions about where the children are physically active and has 3 parts. In the first part, parents are asked to indicate places their children engage in physical activity for more than 10 minutes, and how many days their children have visited the place in the previous 7 days. The places are prespecified and include inside the home, garden/yard, street/streets around the home, organized sport or physical activity, swimming pool/leisure center, community center, religious setting (ie, madrassa, church, or temple), and any other places. The second part asks questions for those of the Muslim faith, and focuses on whether and how often their children have attended a mosque or madrassa in the last 7 days, what time they arrived and left, and whether they actively travelled to and from the mosque or madrassa. These questions were included because the neighborhoods of the JU:MP program have large proportions of Muslim families whose children may attend a mosque or madrassa weekly [27]. The third part includes questions regarding children’s frequency of being active in parks and green spaces (ie, public gardens and playfields). The fifth section of the questionnaire comprises a series of questions asking parents/carers to consider their own parenting practices when it comes to physical activity (autonomy promotion and nondirective support), and how they themselves consider their own neighborhood (neighborhood social cohesion) and the walking/exercise environment characteristics of their neighborhood. These questions are taken from 2 validated questionnaires [44,45]. In the sixth section of the questionnaire, parents/carers will be asked to answer questions regarding their children’s quality of life, which comes from the parent-reported version of the EQ-5D-Y (EuroQol 5-dimension youth version) [46-48] and PedsQL (Pediatric Quality of Life Inventory) [49].

### Sample Size and Power Analysis

A sample size calculation was performed using Stata V.16 (StataCorp), with the function “power two means cluster,” factored in 6 clusters (3 intervention neighborhoods and 3 control neighborhoods), and a 5% 2-sided alpha, as well as an assumed control average daily value of moderate-to-vigorous physical activity of 53.7 minutes, a SD of 19.7, and an intracluster correlation (ICC) value of 0.007, which was conservatively rounded up to 0.01. As the JU:MP project is a neighborhood/community-level intervention and no previous studies using accelerometry as an outcome assessment among children or young people could be identified, the values of average daily moderate-to-vigorous physical activity, SD, and ICC were derived from an unpublished pilot study (sample size n=564; 3 neighborhoods; 12 primary schools). The missingness of data was conservatively factored into the sample size calculation by assuming 30% accelerometer noncompliance at baseline, followed by further 50% loss of data (30% accelerometer noncompliance and 20% attrition) at both the 24-month and 36-month follow-ups. The parameters outlined led to a minimum recommended sample of 1200 children (600 per condition, 200 per neighborhood, and 32 per school) for adequate power (80%) to detect a change of at least 10 minutes in the primary outcome of average daily minutes of moderate-to-vigorous physical activity at both the 24-month and 36-month follow-ups.

### Data Analysis

Data will be entered electronically with a double data entry protocol using REDCAP software. Data will be kept on a secure file storage system, which will also be password protected. Data will be anonymized by the assignment of a unique identification number (study ID) to each participant. The primary comparative analysis (objective 1) will be performed on an intention-to-treat basis, including all participants recruited and without imputation for missing data. Multivariable mixed effects linear regression will be used to estimate differences in the primary outcome (accelerometer-assessed mean daily minutes of moderate-to-vigorous physical activity) between the intervention and control groups. Demographic variables of gender and ethnicity will be treated as random effect variables, and baseline moderate-to-vigorous physical activity, age, accelerometer wear time, BMI z-score, school, neighborhood, and condition will be treated as fixed effect variables. Similar analyses will be repeated for secondary outcomes (objective 2). A sensitivity analysis, using a suitable imputation method, will be conducted to assess the effect of missing data. *P* values and 95% CIs will be calculated. A small number of prespecified subgroup analyses will be carried out to evaluate whether the intervention is differentially effective in different subgroups, such as by neighborhood and school (objective 3). The trial is not powered to detect effectiveness in subgroups, and this analysis will be treated as exploratory, presented using CIs, and interpreted with caution. Mediation analysis using structural equation modeling will be applied to look at whether any intervention effect is mediated by potential determinants (objective 4), including (1) home physical activity, (2) school physical activity, (3) children’s sedentary screen time behaviors, (4) travel and street physical activity, (5) parent practices (nondirective support and autonomy support), (6) neighborhood social cohesion, (7) neighborhood walking/exercise environment, (8) religious setting physical activity, (9) physical activity during sports and recreation, and (10) park/green space physical activity, which the JU:MP program could have impacted based on the complexity of the program. Multimedia Appendix 2, Multimedia Appendix 3, and Multimedia Appendix 4 present 3 directed acyclic graphs that show the simple mediating pathways to be examined, the potential causal pathways between meditating variables, and the potential casual pathways between confounders (sex, ethnicity, age, socioeconomic status, and weight status), respectively.

### Ethical Considerations

This study and all processes were given ethical approval by the University of Bradford Research Ethics Committee (Humanities, Social, and Health Sciences Research Ethics Panel; reference: E891). Informed consent will be sought from schools and parents. Children (participants) will also be asked to provide assent at every data collection timepoint. Schools in the control condition will be paid £600 (US $727) overall (£200 [US $242] for every data collection [baseline, 24-month follow-up, and 36-month follow-up]), and children will be given a “goodie bag” (includes books, stationery, and stickers) worth £2 (US $2.42) as a thank you each time they complete data collection. All data collected will be deidentified and anonymized by using ID numbers and storing all data on secure National Health Service servers, and only the principal investigator and senior researcher will have access to confidential information. All questionnaire data will be double data entered into secure databases created within RedCap.

## Results

This study has been registered at ISRCTN (ISRCTN14332797). Recruitment occurred from July 2021 to March 2022, and baseline data collection occurred from September 2021 to March 2022. As of March 2022 (end of baseline data collection), a total of 1454 children from 37 schools (17 intervention schools and 20 control schools) have been recruited. The first follow-up data collection will occur from September 2023 to March 2024, and the second and final follow-up data collection will occur from September 2024 to March 2025. Data analysis has not begun, and the final results will be published in December 2025.

## Discussion

This paper describes the protocol for a quasiexperimental, nonblinded, nonequivalent group design trial of the JU:MP program, a complex, community, whole-system children’s physical activity intervention. The research outlined in this protocol seeks to explore whether the JU:MP program is effective at increasing the physical activity levels of children aged 5 to 11 years. The recruitment of this trial and baseline data collection have been completed, with 1454 children from 37 schools (17 intervention schools and 20 control schools) being recruited. This number indicates overrecruitment according to the power-calculated target of 600 children per condition (N=1200) and puts the future of this trial in good stead for follow-ups at 24 and 36 months.

Many children and young people do not engage in enough physical activity, with those from ethnic minority backgrounds and economically deprived backgrounds being at greater risk for inactivity and related ill-health. Previous attempts to change children’s physical activity have largely been unsuccessful [15,16]. Moves to more complex interventions using the socioecological model and whole-system approach have increased public health, especially with regard to obesity and physical activity [19,25]. Effectiveness research of whole-system approaches for obesity has been mixed and unclear [50,51]; however, no known effectiveness evaluation has occurred for a whole-system children’s physical activity intervention. The JU:MP program is a complex long-term whole-system intervention (≥2 years) in economically deprived neighborhoods with high proportions of people from ethnic minority groups. The planned and ongoing trial is one of the largest trials ever conducted with an accelerometry outcome measure, which if successful (recruitment and retention rates meet power calculations) will contribute to wider academic public health research by being able to assess the effectiveness of a whole-system approach, which is only currently theorized to be able to change the health of large populations. This effectiveness research is part of a wider body of research with complex process evaluations occurring simultaneously in order to fully examine the full complexity of a whole-system intervention and produce different types of knowledge about a phenomenon that can be combined to further advance knowledge [25,52]

As with all research, there are limitations with the proposed work detailed in this protocol. Namely, the quasiexperimental design of the work naturally has an increased risk of bias when compared with a randomized controlled trial, which is the gold standard way to detect the effects of programs and interventions [53]. However, a randomized controlled trial would not have been feasible for the JU:MP project due to the growing and developing nature of the program in preselected areas of Bradford [25]. Thus, we decided to seek to reduce the risk of bias with matching of neighborhoods based on key descriptive statistics. Another limitation of this work is the reliance on parental/carer and teacher proxy reports, which reduces the validation and reliability of the measures being taken. The reliance on proxy measures is due to the young age of the participants who will not have the capacity to accurately complete questionnaires on key health behaviors (screen time, modes of physical activity, and sleep) and secondary health outcomes such as quality of life and emotional well-being. However, the use of accelerometry as an outcome assessment overcomes the capacity issue of younger children and provides a more objective tool to measure the complex behavior of physical activity.

Many attempts have been made by researchers, practitioners, and policy makers to address children’s physical inactivity. However, previous attempts have been unsuccessful in both intervention design and the application of complex evaluations. Lack of success leads to children’s physical activity levels stagnating or worsening, increasing the risk of ill-health and negatively impacting the growth and development of young people. Sport England, the largest funder of sports and physical activity in England, has invested significant funds in 12 geographical areas in England to increase physical activity through a place-based whole-system approach. Methodologically rigorous and high-quality research is required to examine what works, why, for whom, and in what context, in order to understand the potential of whole-system approaches for increasing children’s physical activity, and whether and how they can be replicated [25]. Research evaluating one of the geographical areas (the JU:MP project in the City of Bradford) seeks to achieve this endeavor. This protocol outlines the effectiveness part of the evaluation.

## Appendix

Multimedia Appendix 1 Join Us: Move Play (JU:MP) neighborhood map. A geographical map of the JU:MP neighborhood boundaries within the local delivery pilot area. The depicted map is adapted from the QGIS Geographic Information System (2021) and Open Source Geospatial Foundation Project.

Multimedia Appendix 2 Directed acyclic graph showing the potential casual pathways between mediators and the outcome. JU:MP: Join Us: Move Play; MVPA: moderate-to-vigorous physical activity; PA: physical activity.

Multimedia Appendix 3 Directed acyclic graph showing the potential casual pathways between mediators and the outcome, with interconnections between mediators. JU:MP: Join Us: Move Play; MVPA: moderate-to-vigorous physical activity; PA: physical activity.

Multimedia Appendix 4 Directed acyclic graph showing the potential casual pathways between mediators and the outcome, with possible casual pathways between confounders. JU:MP: Join Us: Move Play; MVPA: moderate-to-vigorous physical activity; PA: physical activity; SES: socioeconomic status.

## Abbreviations

ICC: intracluster correlation
IMD: index of multiple deprivation
JUMP: Join Us: Move Play
SDQ: strengths and difficulties questionnaire
YAP: youth activity profile

## Data Availability

This is not applicable at present as this is a protocol article. However, future and final data sets will be available from the corresponding author and a Born in Bradford (BiB) executive on reasonable request. Scientists are encouraged and able to use BiB data, which are available through a system of managed open access. The steps below describe how to apply for access to BiB data.

Before you contact BiB, please make sure you have read our guidance for collaborators. Our BiB executive reviews proposals on a monthly basis, and we will endeavor to respond to your request as soon as possible. You can find out about the different data sets available. If you are unsure if we have the data that you need, please contact a member of the BiB team. Once you have formulated your request, please complete the “Expression of Interest” form available and send it to borninbradford@bthft.nhs.uk. If your request is approved, we will ask you to sign a collaboration agreement, and if your request involves biological samples, we will ask you to complete a material transfer agreement.
