# Supplementary figures and images for: Evaluation of the Effectiveness of a Whole-System Intervention to Increase the Physical Activity of Children Aged 5 to 11 Years (Join Us: Move Play, JU:MP): Protocol for a Quasiexperimental Trial
Source: JMIR Res Protoc. 2023 Mar 31;12:e43619. doi: 10.2196/43619 (PMC10131718; doi:10.2196/43619)

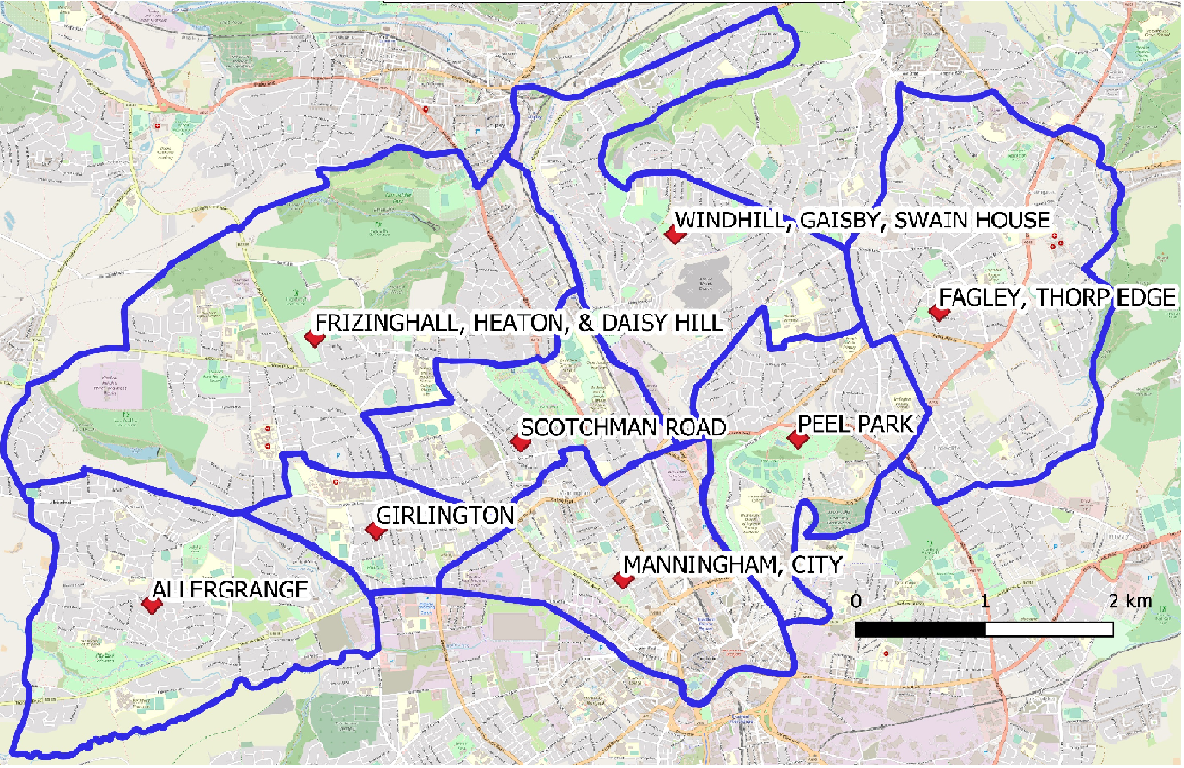

Supplement: Multimedia Appendix 1 [file resprot_v12i1e43619_app1.png]

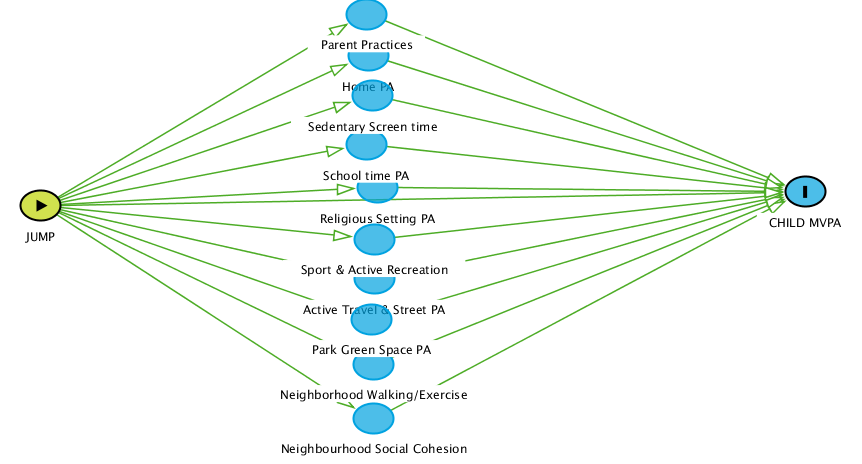

Supplement: Multimedia Appendix 2 [file resprot_v12i1e43619_app2.png]

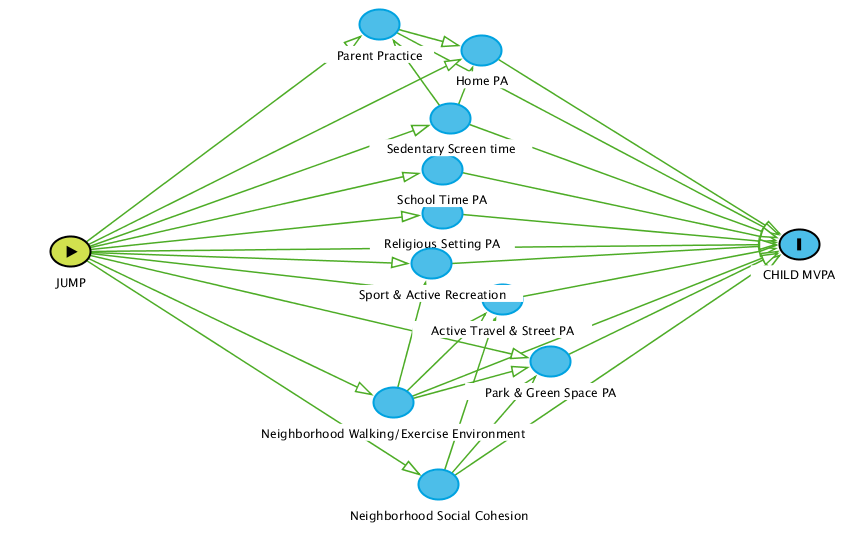

Supplement: Multimedia Appendix 3 [file resprot_v12i1e43619_app3.png]

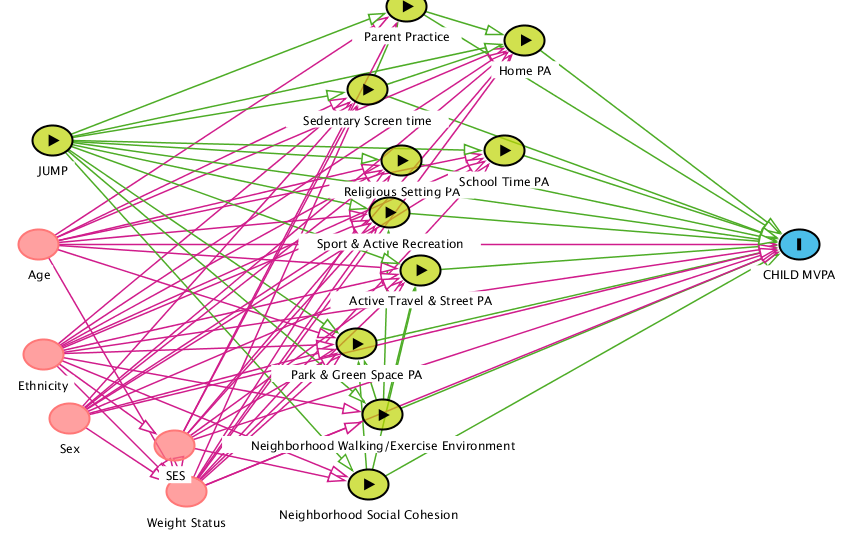

Supplement: Multimedia Appendix 4 [file resprot_v12i1e43619_app4.png]
